# The Role of Alternative Crops in an Upcoming Global Food Crisis: A Concise Review

**DOI:** 10.3390/foods11223584

**Published:** 2022-11-11

**Authors:** Antonios Mavroeidis, Ioannis Roussis, Ioanna Kakabouki

**Affiliations:** Laboratory of Agronomy, Department of Crop Science, Agricultural University of Athens, 11855 Athens, Greece

**Keywords:** alternative crops, Global Food Crisis, Food Security, Sustainable Development Goals

## Abstract

Achieving Food Security (FS) is perhaps our most challenging aspiration. Despite our best efforts, millions of people around the globe are malnourished or live with hunger. The state of the geo-political scene, as well as the COVID-19 pandemic, have recently brought forth fears of a Global Food Crisis (GFC). Here, we present the factors that threaten FS and could trigger a GFC, examine the potential of alternative crops (ACs) as a measure against an upcoming GFC, and highlight the key aspects of the ACs introduction process in new regions. ACs could enhance FS, yet their success is premised on the adoption of sustainable practices and the implementation of food strategies that aim to promote healthy consumer behaviours.

## 1. What Makes a Food Crisis?

Since the dawn of mankind, hunger has been our omnipotent enemy. From simple food shortages to famines, whether on a national, regional, or global scale, human history is filled with hunger. By the late 1800s, Walford had recorded more than 70 famines during the 18th–19th centuries throughout Europe, Asia, Africa, and South America [1]. Following the Green revolution of the 1950s–1960s, the frequency, intensity, and mortality of famines were drastically reduced [2]. Nevertheless, malnutrition still affects at least 200 million people [3]. Nowadays, the term “food crisis” is frequently being used when discussing the malnutrition conundrum. Based on the definition by Timmer [4], a food crisis is “the sharp rise of hunger and malnutrition rates at local, national, or global levels”. Recently, mainly due to the COVID-19 pandemic and the invasion of Ukraine, fears of an upcoming global food crisis (GFC) have arisen [5,6].

Any food crisis could be perceived as a complicated nexus of socioeconomic and environmental factors. Broadly speaking, any phenomenon that threatens food security (FS) could constitute a driving force behind a food crisis (i.e., armed conflicts, economic busts, and climate change). Being a major threat to agriculture, climate change has increased food insecurity globally. In a study by Dasgupta and Robinson [7], the authors concluded that for every 1 °C of temperature increase, severe global food insecurity was raised by 1.64% during 2019. The findings of this study also suggested that countries with weak economies are particularly susceptible to climate change-induced food insecurity [8]. Besides the increasing temperature, droughts are expected to become more frequent and more intense [9], and soil salinization could be exacerbated, especially in dryland areas [10]. Climate shifts might increase insect and pathogen pressure and reduce pesticide efficacy [10,11].

Climate change mitigation would decrease the chances of a food crisis. However, designing an effective mitigation policy could be tricky. According to Hasegawa et al. [12], the implementation of a horizontal and strict mitigation policy could destabilize the prices and the supply chains of key agricultural commodities, and thus dent global food security. Such destabilizations are known to boost malnutrition rates. In fact, the relatively recent GFC threat we faced during 2007–2008 was heavily attributed to the elevated prices of basic food products [4,13]. Disturbingly, food prices have once again skyrocketed. The update on the World Food Situation, released by the FAO on 8 April 2022, reported an all-time high record of the Food Price, Cereal Price, Vegetable Oil Price, and Meat Price Indices [14]. The incremental tendency of these FAO indices was anticipated following the invasion of Ukraine. Nonetheless, prior to the invasion, or even the outbreak of COVID-19, the existing climate scenarios predicted that the prices of wheat, maize, and rice would increase by more than 30% by 2050 [15]. The pandemic only deteriorated the situation, as it caused perhaps the most severe post-WWII economic downturn [6].

**Figure 1:**
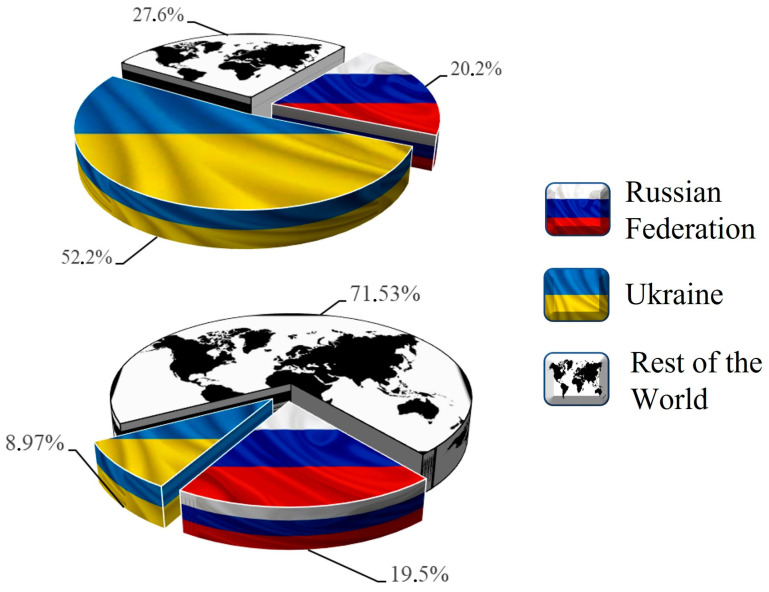
The share of Ukraine, the Russian Federation, and the rest of the world in the global exports of sunflower-seed or oil (crude) on the top, and wheat on the bottom, during 2021. Data obtained from the official website of the Observatory of Economic Complexity (OEC) (https://oec.world/en) (accessed on 14 October 2022).

Concurrently, the Russo-Ukrainian conflict is considered by many a food-security ticking bomb [16]. War is a major, if not the main, driver that pushes people to malnutrition and hunger. According to the latest report on GFC by the Global Network Against Food Crises (GNAFC), during 2021, armed conflicts drove nearly 140 million people from 24 countries/territories into food crisis [3]. However, the invasion of Ukraine is more than an armed conflict. Both the Russian Federation and Ukraine are major net exporters of agricultural products, and leading suppliers of agro-food commodities in the global markets [17] (Figure 1). Notably, in 2021 these two countries were amongst the top global exporters of wheat, maize, rapeseed, sunflower seeds, and sunflower oil [17]. In the same year, the Russian Federation was the top, second, and third largest exporter of nitrogen, potassium, and phosphorus fertilizers, respectively [17]. The conflict between these two countries could disrupt the global food, fertilizer, and fuel systems and supply chains, plunging millions into hunger [16].

On top of all that, the world is amidst the COVID-19 pandemic, which impaired agricultural production [6] and economic activities [18]. As lockdowns came into force all around the globe, transportation restrictions led to manpower scarcity on a farm level [6]. This was also the case for the food-processing industry, as gradually more and more workers contracted COVID-19 and food-industry plants were forced to temporarily halt production or operate at much slower rates [19]. Food trade restrictions negatively affected food distribution [19]. Once again, countries with weaker economies, poor healthcare systems, and labor-intensive agricultural sectors were found to be less resilient to the COVID-19-induced shocks to agriculture and to food-supply chain disruptions [6,19].

Some might argue that malnutrition mainly affects the developing countries and that the countries of the first world are not in an immediate threat of a food crisis. For instance, despite the aforementioned information, the European Union (EU) is self-sufficient in the majority of key agricultural commodities, and the availability of food within the EU is probably not at risk [20]. However, the accessibility of food prices, as well as the availability of fertilizers and animal feed, are still open to doubt [20]. Based on the above, the status quo is at least alarming as the threat of a GFC is lurking in the horizon. It is possible that we are already in the midst of such a crisis. In the 2022 Global Report on Food Crisis, the Secretary-General of the United Nations stated that “we are facing hunger on an unprecedented scale, food prices have never been higher, and millions of lives and livelihoods are hanging in the balance” [3]. Of course, managing such a threat is a convoluted task that requires coordinated interdisciplinary collaborations. Here, we will focus on the potential, strengths, and weaknesses of alternative crops.

## 2. Alternative Crops and Food Security

Initially, we need to define what an alternative (or novel, innovative, retrovative, etc.) crop is. In most cases, the alternative crops (ACs) are described as crops that can be introduced into a new agroecosystem in lieu of “traditional” crops that are usually more susceptible to biotic (e.g., pests) and abiotic stress [21] (e.g., salinity). For instance, heritage cereals that are usually more resilient to extreme weather events compared to the modern cereal varieties [22,23], could be characterized as ACs. According to Isleib [24], ACs are crops (re)introduced in a particular geographic area due to their potential high value or other benefits to the farming systems of that area. ACs are frequently mixed up with the underutilized species (NUS) (also known as neglected, orphan, or niche crops) [25]. NUS were primarily being cultivated in their center of origin; however, at some point in time they lost favor and now have regained interest (locally or in a wider scale) [26]. The concepts of ACs and NUS have apparent differences, yet some NUS could be perceived as ACs, provided they have been proposed as promising crops to be (re)introduced to an area of adaptation. Subsequently, there is no strict classification of a group of crops as ACs (Table 1). As a case in point, teff is regarded as an AC in the Mediterranean Basin [27], yet as a traditional crop in Ethiopia [28].

In order to evaluate the potential role of ACs in an upcoming GFC, initially we have to examine their beneficial effects on food security (FS). According to the definition by FAO, FS is the state when “all people have physical and economic access to sufficient, safe, and nutritious food that meets their dietary needs and food preferences for an active and healthy life” [29]. Of course, “access” is not the only factor that defines FS. FS is founded on four pillars: availability, access, utilization, and stability.

The availability of food is both a quantitative, and a qualitative indicator, as it refers to the existence of sufficient amounts of domestically produced and/or imported nutritious food [30]. To enhance food availability, the introduction of an AC to a region should aim to increase the quantities of produced food commodities in that region, and offer high-quality, nutritious food alternatives. Several crops rich in micro- and macronutrients have been proposed as ACs (Table 1). The literature also highlights the acclimatization and adaptation potential of some of them under high salinity, drought conditions, water logging, and in soils with low fertility [26,27]. The introduction of ACs to such low-productivity areas could increase food availability.

**Table 1:** List of crops that have been proposed as ACs [21,27], their common and scientific names, family, area of origin, and nutritional value. These crops have been proposed as ACs due to their acclimatization potential to marginal environments and/or their tolerance to biotic (pests and diseases) or abiotic stress (high salinity and sodicity, droughts, and high temperatures).

Common Name	Scientific Name	Family	Area of Origin	Nutritional Value	Referance
Amaranthus	Amaranthus retroflexus	Amaranthaceae	Americas	Seeds abundant in protein content (13–19%), high levels of oils rich in squalene, and high amounts of antioxidants	[31]
Buckwheat	Fagopyrum esculentum	Polygonaceae	Asia	Protein content similar to that of wheat, aproximantely 3% fat content, and high crude fiber concentration	[32]
Canihua	Chenopodium pallidicaule	Amaranthaceae	Andes	Exeptional protein, fat, ash, and carbohydrate content	[33]
Einkorn	Triticum monococcum	Poaceae	Asia Minor	Rich in antioxidant compounds such ascarotenoids, tocols, conjugated polyphenols, alkylresorcinols, and phytosterols	[34]
Emmer wheat	Triticum dicoccon	Poaceae	Eurasia	Rich in resistant starch, minerals, fibre, carotenoids, and antioxidant compounds	[35]
Foxtail	Setaria italica	Poaceae	Southern Asia	Rich in protein, fatty acids, minerals, and amino acids	[36]
Khorosan wheat	Triticum turgidum ssp. Turanicum	Poaceae	Mesopotamia	Higher protein, crude ash, and vitamine E content compared to wheat	[37]
Pearl millet	Cenchrus americanus	Poaceae	West Africa	360 calories, 12 g of protein, 5 g of fat, 1 g of fibres, and 67 g of carbohydrates per 100 g of seeds	[38]
Quinoa	Chenopodium quinoa	Amaranthaceae	Andes	Gluten-free, with high protein concent, rich in unsaturated fatty acids, vitamins, and minerals	[39]
Salicornia	Salicornia bigelovii	Amaranthaceae	North America	Rich in bioactive compounds, vitamin A, minerals and fatty acids. Seedoil rich in linoleic acid	[40]
Spelt	Triticum spelta	Poaceae	Europe	Higher protein content, more non-essential amino acids, and less lysine	[41]
Tef	Eragrostis tef	Poaceae	Somali Peninsula	Gluten-free, rich in protein, dietary fiber, polyphenols, and minerals	[42]
Triticale	×Triticosecale	Poaceae	Europe	High protein content and slightly higher levels of most of the nutritious compounds when compared to wheat	[43]
Tritordeum	Tritordeum martinii	Poaceae	Europe	High total phenol content, antioxidant activity, dietary fiber content, and total free amino acids	[44]
Cowpea	Vigna unguiculata	Fabaceae	Southern Africa	Rich in protein (<20%) and minerals (calcium, potassium, sodium, and more)	[45]
Guar	Cyamopsis tetragonolobus	Fabaceae	Africa	High protein, ash, and polyphenol contents	[46]
Lablab	Lablab purpureus	Fabaceae	South-east Asia	Rich in proteins, carbohydrates, minerals and vitamins	[47]
White lupin	Lupinus albus	Fabaceae	Mediteranean Basin	Fair protein, fatty acid, and fibre content, as well as oligosaccharides, antioxidants, and non-starch carbohydrates	[48]
Pigeon pea	Cajanus cajan	Fabaceae	South Asia	Rich in starch, protein, calcium, manganese, crude fibre, fat, and minerals	[49]
Sesbania	Sesbania sp.	Fabaceae	North-East Africa	High protein content (can exced 40%), vitamin C, and calcium	[50]
Indian mustard	Brassica juncea	Brassicaceae	West Asia	Seeds rich in glucosinolates, sterols, and phenols. Leafs rich in glucose, fructose, and minerals	[51,52]
Purslane	Portulaca oleracea	Portulacaceae	Eurasia	Rich in omega-3, amino acids, and vitamins	[53]
Chia	Salvia hispanica	Lamiaceae	Central America	Seeds with high protein content (>15%), rich in lipids, and minerals. On average, 100 g of seed contains aproximantely 500 kcal	[54]
Nigella	Nigella sativa	Ranunculaceae	Eastern Europe	Rich in fatty acids, phytosterols, glycolipids, and phospholipids	[55]
Sweet potato	Ipomoea batatas	Convolvulaceae	Americas	Protein content ranging from 4–27%, rich in β-carotene and anthocyanin	[56]
Camelina	Camelina sativa	Brassicaceae	Europe	Excelent source of essential unsaturated fatty acids, particularly OMEGA-3 fatty acids	[57]

As marginal areas constitute a significant portion of available land, their exploitation via the introduction of stress tolerant ACs would increase their agricultural productivity, and thus enhance FS [21]. For instance, in Iran, high salinity and droughts are major obstacles to agriculture [58]. The experimental incorporation of quinoa, a salinity tolerant crop, in Iran reported promising results as irrigation with 14 dS m^−1^ of saline water resulted in grain yields of 2–3 t/ha [21].

Access to food is mainly determined by economic factors [59]. Typically, the balance between food prices and household income/assets influences food access. The prices of agricultural commodities usually depend on their supply and demand [60]. Shifts in their supply/demand equilibrium tend to alter their price. This affects FS, as extreme downward or upward price oscillations have been proven to be detrimental to food access [61]. From a financial point of view, ACs can boost food access to rural areas by improving the agriculture household income, especially in the case of small- and medium-scale farmers [62]. Moreover, ACs promote food/crop diversity. Crop diversification can stabilize the farmers’ flow of income, especially in a small-farm scale [63]. Additionally, they provide resilience to income shocks and opportunities for improved incomes, due to the potentially favorable prices of ACs [64,65,66].

The FAO defines food utilization as “the proper biological use of food”, under the context of a healthy diet that provides sufficient energy and essential nutrients [67]. Modern agriculture relays heavily on a few staple food crops to meet the global demand. It is estimated that wheat, maize, and rice provide more than half of the world’s plant-derived calories [26]. That being the case, a lack in dietary variety can lead to malnutrition despite adequate caloric intake [59]. Enriching crop diversity would benefit both the FS and the natural agroecosystems [68]. The introduction of ACs could increase the versatility and improve the nutritional content of meal plans, as the grains of several ACs are rich in proteins, fats, crude fibers, micro- and macronutrients, etc. [69]. Moreover, crop diversity is known to benefit nutritional stability [70]. Admittedly, due to their poor market presence, the advancements in processing and storage methods of AC final products are often lackluster [26]. Proper storage is essential for tackling food insecurity [71].

Food stability can be achieved when food availability, access, and utilization are consistent through time [29]. This dimension of FS comprises an expression of the need for sustainable food production and sustainable agricultural systems. Sustainable agriculture itself is compromised of the changing climate, the loss of agricultural biodiversity, soil degradation, and water and air pollution [72]. The intensification of agriculture and the mainstay inputs of conventional agricultural systems only aggravate the situation [73]. On the contrary, the adoption of ACs could tackle these constraints on sustainable agriculture. Besides the enhancement of crop diversity, many ACs often require low (compared to traditional crops) chemical inputs in the form of fertilizers and pesticides [27]. Similarly, the drought tolerant ones are characterized by reduced irrigation needs [27]. Due to their low input needs, they can perform adequately under organic systems [27]. As a result, the introduction and cultivation of ACs could further reduce environmental degradation. In a recent study by Mazac et al. [74], the authors estimated that the incorporation of novel foods in European food systems would contribute to global warming mitigation, as well as improve water and land use by over 80%.

## 3. Food for Thought

The introduction of ACs should be dealt with caution, otherwise, not only will they not contribute to FS, but they could also be unprofitable for the farmers and damaging to the agricultural systems they are introduced into. To fully understand this dynamic, one can refer to the example of quinoa. Quinoa originates from the South American Andes, where it has been cultivated for more than 8000 years [75]. Following the 1950s, quinoa gained international attention, due to its high nutritional value, that peaked around the mid-2010s [76]. As the demand for quinoa grew, the crop was introduced to many countries in Europe and North America, though they continued to import significant amounts of quinoa from the three major producers (Peru, Bolivia, and Ecuador) [75,76]. Quinoa markets boomed, the demand increasing rapidly, and the international prices were elevated. In the Andes, the attractive prices of quinoa shifted its cultivation from small-farm “traditional” models to large-scale, market-oriented farming [75]. Cultivation of quinoa was intensified to the point that land use changes, land degradation, extensive monocropping, and the loss of genetic diversity threatened the sustainability of both the production of quinoa and the local agroecosystems [75,76]. Soon, the production of quinoa in the Andes doubled, the supply of quinoa exceeded the global demand, and the prices fell [76]. This phenomenon is known as “boom and bust”, a chain reaction catalyzed by market trends, that results in acute price oscillations and a shift towards less sustainable agricultural practices [76].

**Figure 2:**
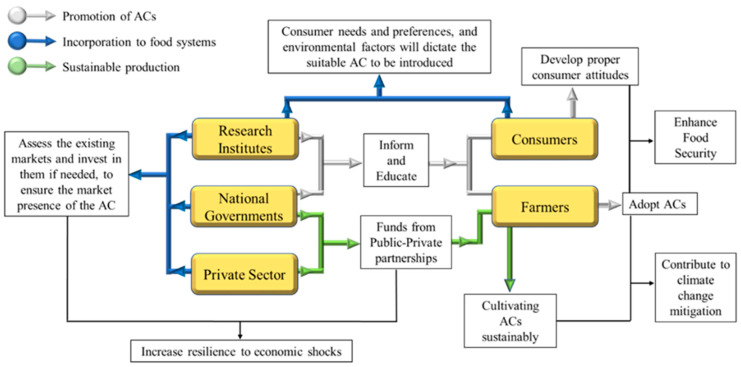
Highlights of the three stages of introducing ACs. All the actors (yellow color) of the ACs value chain and their key interactions are briefly depicted in the figure. The actions and interactions of each stage are depicted with different colors (white, blue, and green). As the three stages are not necessarily successive, actions and interactions of the actors from different stages could be simultaneous.

Andreotti et al. [76] acknowledged that the “boom and bust” of such crops can be divided in stages: promotion, boom, bust, and transition to a new system. These stages could function as the pillars upon which policymakers can design frameworks for the introduction of ACs to new areas. Here, we will highlight key features of the ACs integration process that, based on the literature, as well as empirical knowledge, should always be regarded. Based on the work of Andreotti et al. [76], this process could be divided into three phases: promotion of ACs, incorporation to food systems, and sustainable production, and they should aim to avoid the “boom and bust” phenomenon (Figure 2).

Promotion of ACs: Promoting an AC is based on the simple, yet admittedly challenging task of raising awareness and educating the public. This can be done via mainstream and social media, workshops and living labs, educational initiatives, or even peer-to-peer interactions. National governments possess the means (e.g., taxations and subsidies) to motivate farmers to adopt ACs. Farmers on their behalf should comprehend every beneficial aspect of the ACs on FS and sustainable agricultural practices, instead of focusing solely on their potential short-term economic returns. This tendency has also been observed on the adoption rates of integrated pest management strategies [77]. However, well-informed farmers have been reportedly more likely to change their attitudes towards these practices [78]. Well-informed farmers might also be more likely to adopt ACs. Consumers, on the other hand, might already be more willing to embrace ACs. A recent study by Wendin et al. [22] found that, in the case of heritage cereals, women and elders amongst the different age/sex groups were the most concerned regarding the origin and health benefits of the AC, and the elderly were more willing to pay higher prices for the AC products. They also reported that in the majority of the participants in their study were aware of the heritage cereals (to least at some extent). The authors attributed this finding partially to the recent health trends that have been related with such ACs [22]. However, consumers (mainly in developed countries) should adopt proper attitudes and not simply follow food trends.

At this point, a paradox needs to be addressed. As mentioned above, nearly 200 million people are currently in food crisis all over the world [3]. Yet more than 700 million are on the opposite side of the nutritional spectrum, being overweight or obese [79]. The rates of obesity are expected to increase, and by 2030, more than 1 billion people are estimated to be living with obesity [79]. Obesity is rapidly turning into an epidemic, especially in the developed countries. Recent studies report that more than 40% of North Americans are obese and approximately 60% of Europeans are either overweight or obese [80,81]. The literature strongly suggests that, besides the plethora of health problems that have been attributed to obesity, there is a link between it and COVID-19 high mortality rates [82]. As health experts call for a solution, the timing is perfect for proposing more diverse and healthy diets. ACs could offer viable solutions to meal plans and their promotion could be part of a healthier “new food agenda”, that could also target younger audiences (e.g., inclusion of zero food waste and healthy diet-related lectures in school curriculums) and help them develop healthy eating habits from their early years.

Incorporation into food systems: Initially, the selection of the introduced AC is premises on meticulous planning based on region-specific studies. Several factors must be considered, including environmental and pedoclimatic niches, regional food preferences and needs, and the societal benefits of the AC to local communities. The involvement of National Agricultural Research Systems (NARS) is vital. Research should also include breeding programs to improve the crop if needed (e.g., reduce seed heterogeneity). However, the importance of the genetic diversity of the crop should not be neglected, as it relates to the crop’s adaptability [75]. The lesson learned from quinoa’s boom and bust is that when ACs transition from smallholding to an industrial agriculture model, the crops’ genetic variety gets disregarded, due to market pressure [75]. The industrialization of the ACs might also ignore the empirical farming knowledge passing down from generation to generation (especially in the case of NUCs). Ex situ gene banks and the utilization of cultivation-practices related to traditional knowledge will be essential for the improvement of the ACs’ performance [26]. Finally, it is crucial to ensure the ACs market presence. This requires the assessment of the dynamics of regional agricultural development, the existing markets and supply chains, and the logistics costs.

Sustainable production: Research on the optimization of cultivation practices (e.g., fertilization, irrigation, etc.) and food processing should be constant and not limited during the introduction of ACs in a new food system. The effects of ACs on the environment and on the everyday lives of rural populations should be regularly evaluated. Governments, civil society organizations, and private partners should monitor the value chains of ACs and interfere when needed to avoid any boom-and-bust scenarios. Overall, the cooperation of both the public and the private sectors (public-private partnerships) would be beneficial for the sustainability of ACs, and the agricultural systems as a whole. A recent report by the UN Food Systems Summit, the World Bank, the International Food Policy Research Institute (IFPRI), and the Food and Land Use Coalition presented the Food Finance Architecture, a five food finance imperatives-based policy for sustainable food systems [83]. Under this context, governments and private sector partners could mutually finance investments with social and environmental impact, such as the incorporation of ACs in food systems.

**Figure 3:**
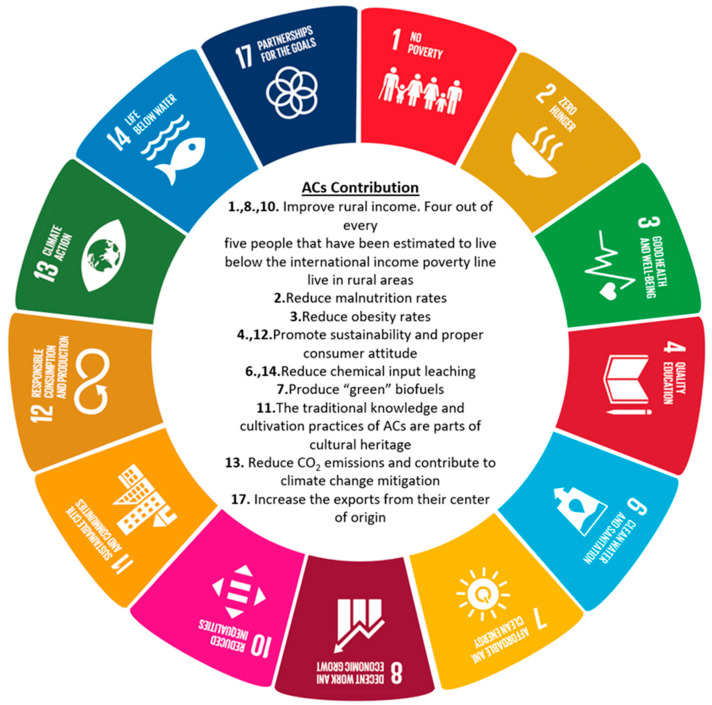
ACs and the SDGs set by the UN. Each SDG is depicted with a different color and is attributed its corresponding number. The contribution of ACs to the SDGs is depicted in the center of the figure next to the corresponding number of each SDG.

ACs are very promising for the future of agriculture. After all, the introduction of ACs also complies with the Sustainable Development Goals (SDGs) set by the UN (Figure 3). The sustainability of agriculture has become a major challenge for policy makers all around the globe. Both the EU and the USA, two of the most significant economic regions of the world with vastly contrasting approaches in agriculture, have designed their strategies to achieve that [84]. ACs seem to be fitting for the Special Objectives of the EU’s Common Agricultural Policy 2023–2027 [27] (climate change mitigation, biodiversity enhancement, sustainable food production), the aims of the European Green Deal [27] (reduction of chemical inputs, creation of sustainable food labeling, reduction of greenhouse gases emissions), as well as the aspirational goals of the US Agriculture Innovation Strategy [85] (market expansion and diversity). The adoption of ACs could also facilitate the implementation of the Agenda 2063 that aims to enhance Africa’s collective FS by 2063 [86], and the 2030 Strategy of the Asian Development Bank that intends to tackle climate change while strengthening FS in Asia by 2030 [87]. ACs can be grown sustainably, but at the same time, they have much to offer to the concept of agricultural sustainability itself.

## Data Availability

Not applicable.

## Abbreviations

GFC, Global Food Crisis; FS, Food Security; FAO, Food and Agriculture Organization of the United Nations; GNAFC, Global Network Against Food Crises; EU, European Union; ACs, Alternative Crops; NUCs, Neglected and Underutilized Crops; NARS, National Agricultural Research Systems; UN, United Nations; IFPRI, International Food Policy Research Institute; SDGs, Sustainable Development Goals; US, United States.
